# Effect of Different Local Vibration Frequencies on the Multiscale Regularity of Plantar Skin Blood Flow

**DOI:** 10.3390/e22111288

**Published:** 2020-11-13

**Authors:** Fuyuan Liao, Keying Zhang, Lingling Zhou, Yanni Chen, Jeannette Elliott, Yih-Kuen Jan

**Affiliations:** 1 Department of Biomedical Engineering, Xi’an Technological University, Xi’an 710021, China; liaofuyuan1024@163.com; 2 Rehabilitation Engineering Laboratory, Department of Kinesiology and Community Health, University of Illinois at Urbana-Champaign, Champaign, IL 61820, USA; keyingz@illinois.edu (K.Z.); lz18@illinois.edu (L.Z.); 3 Department of Pediatrics, Xi’an Jiaotong University Health Science Center, Xi’an 710021, China; chenyannichil@163.com; 4 Disability Resources and Educational Services, University of Illinois at Urbana-Champaign, Champaign, IL 61820, USA; elliott3@illinois.edu

**Keywords:** local vibration, multiscale entropy, skin blood flow, vibration frequency, regularity

## Abstract

Local vibration has shown promise in improving skin blood flow (SBF). However, there is no consensus on the selection of the best vibration frequency. An important reason may be that previous studies utilized time- and frequency-domain parameters to characterize vibration-induced SBF responses. These parameters are unable to characterize the structural features of the SBF response to local vibrations, thus contributing to the inconsistent findings seen in vibration research. The objective of this study was to provide evidence that nonlinear dynamics of SBF responses would be an important aspect for assessing the effect of local vibration on SBF. Local vibrations at 100 Hz, 35 Hz, and 0 Hz (sham vibration) with an amplitude of 1 mm were randomly applied to the right first metatarsal head of 12 healthy participants for 10 min. SBF at the same site was measured for 10 min before and after local vibration. The degree of regularity of SBF was quantified using a multiscale sample entropy algorithm. The results showed that 100 Hz vibration significantly increased multiscale regularity of SBF but 35 Hz and 0 Hz (sham vibration) did not. The significant increase of regularity of SBF after 100 Hz vibration was mainly attributed to increased regularity of SBF oscillations within the frequency interval at 0.0095–0.15 Hz. These findings support the use of multiscale regularity to assess effectiveness of local vibration on improving skin blood flow.

## 1. Introduction

Foot ulcerations is some of the most common complications in people with diabetes mellitus [1,2]. The recurrence rate of foot ulcers is estimated at about 40% within 1 year, 60% within 3 years, and 65% within 5 years [1]. The etiology of diabetic foot ulcers involves a number of factors, including peripheral neuropathy and peripheral arterial disease [3,4,5]. Peripheral neuropathy causes a series of pathologic alterations in the foot, such as loss of protective sensation for perceiving mechanical stresses, foot deformity, and skin dryness. Peripheral arterial disease causes an impaired vasodilatory response to repetitive plantar pressure during walking. Therefore, interventions that can improve skin blood flow (SBF) and reduce plantar ischemia are needed to prevent foot ulcers in at-risk patients [1,2,5]. 

Local vibration has shown promise in increasing SBF, reducing tissue ischemia and improving wound healing [6,7,8,9,10,11,12,13]. However, there is no specific guideline on the selection of the appropriate vibration frequency due to conflicting results. Different studies have used different vibration frequencies without any rationale for choosing the frequency. Corbiere and Koh [7] applied 90 Hz vibrations to the feet of mice and observed improved healing of muscle injury. For human participants, Maloney-Hinds et al. [8] applied both 30 Hz and 50 Hz vibrations to the forearm of healthy adults for 10 min, respectively. The results showed that both vibration frequencies caused significant increases in SBF; and 50 Hz vibration was more effective than 30 Hz. Ren et al. [10] compared the effect of different intermittent durations of local vibration on SBF at the middle metatarsal head of diabetic and healthy adults using 50 Hz frequency. Zhu et al. [11] investigated the preconditioning effect of 100 Hz vibration on plantar SBF response to walking and observed reduced walking-induced hyperemic response. Zhu et al. [12] investigated plantar SBF responses induced by different vibration frequencies. They applied three frequencies of vibration (i.e., 0 Hz (sham vibration), 35 Hz, and 100 Hz) at the first metatarsal head of healthy adults. The results showed that 100 Hz vibration significantly increased SBF compared to 35 Hz and 0 Hz vibrations. The lack of consensus on the effect of vibration frequency on SBF could be due to an insufficient understanding of the SBF response to vibration. The use of mean value of SBF responses to vibration may not fully characterize the effect of vibrations on the microvascular system [14,15]. 

The regulation of the SBF response to mechanical stress is a dynamic process, involving changes not only in blood flow values, but also in the structural properties (dynamics) of SBF [14,15,16,17]. In the literature, the use of time- and frequency-domain based analyses cannot fully capture the effect of local vibrations on SBF responses. Time-domain parameters provide intuitive features for SBF regulation, for example, an increase or decrease in SBF after a stress. Spectral analyses in the frequency domain provide methods to study SBF control mechanisms [18,19]. However, these time- and frequency-domain analyses are unable to characterize the structural properties of SBF, e.g., complexity and self-similarity [20]. Our previous studies have demonstrated that altered non-linear properties of SBF are associated with impaired microvascular function [16,17,20,21,22]. 

The objective of this study was to investigate the structural properties of SBF in response to local vibrations at different frequencies. Specifically, a modified sample entropy algorithm [21] was used to quantify the multiscale regularity of SBF. We hypothesized that different frequencies of local vibration would cause different changes in the multiscale regularity of SBF. To the best of our knowledge, this is the first study investigating the effect of local vibration on the multiscale regularity of SBF. 

## 2. Methods

A repeated measures study design was used to compare the effect of three frequencies of local vibration on SBF responses. This study was part of a large study [12]. The data of this study have not been reported before. 

### 2.1. Participants

Twelve healthy subjects were enrolled in this study. The inclusion criteria were healthy adults aged between 18 and 35 years. The exclusion criteria included cardiovascular diseases, diabetes mellitus, skin diseases, and neurological diseases as well as taking any medication that may affect microvascular function. This study was approved by the University of Illinois at Urbana-Champaign, Institutional Review Board (#20322). All participants signed the informed consent forms before any tests. Their demographic data were: were (mean ± standard deviation): age 25.4 ± 5.5 years, height 1.65 ± 0.5 m, weight 60.3 ± 7.6 kg, body mass index (BMI) 22.0 ± 2.4 kg/m^2^, diastolic blood pressure 68.5 ± 7.4 mmHg, systolic blood pressure 105.1 ± 14.4 mmHg, and heart rate 67.9 ± 6.4 bpm. 

### 2.2. Experimental Procedures

**Figure 1:**
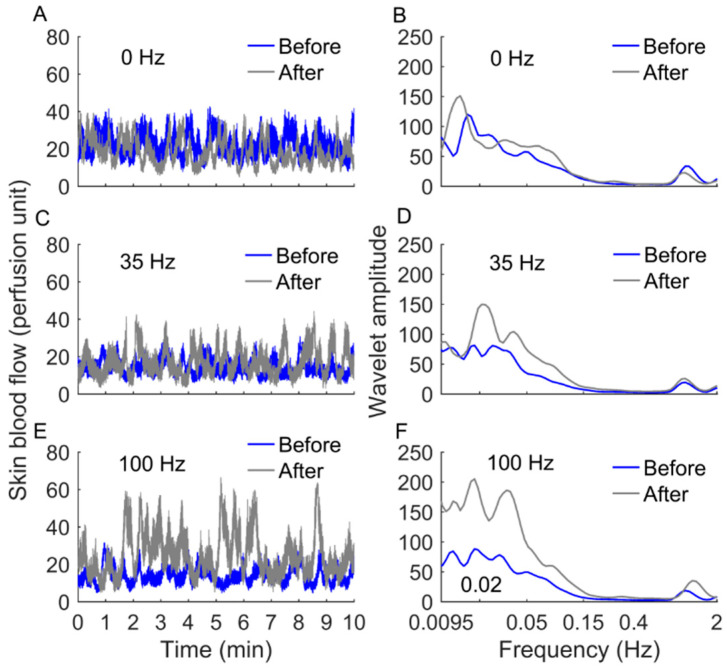
Skin blood flow signals and their wavelet spectra of a subject before and after local vibration at 0 Hz (**A**,**B**), 35 Hz (**C**,**D**), and 100 Hz (**E**,**F**).

The experiments were conducted in a laboratory where the temperature was maintained at 24 ± 2 °C. Prior to the tests, the subject rested in the lab for at least 30 min to achieve a stable level of SBF. During the experiment, the subject lay in the supine position on a table with the right leg and foot being supported by a custom-designed frame to avoid possible movement as well as fatigue. An assembled vibrator was used to apply local vibration at three frequencies of 0 Hz (sham vibration), 35 Hz, and 100 Hz with the amplitude of 1 mm to the right first metatarsal head in a random order [12]. This device consisted of a voice coil motor (YLM40-20, JDStek, Los Angeles, CA, USA), a controller (MS 15 TTLx20, RSF Elektronik, Tarsdorf, Austria), and a power supply, with capacity to generate vibrations at adjustable frequency and amplitude. The vibrator head was custom designed, made of thermoplastic elastomer materials, in a cylinder shape with a diameter of 20 mm. The selection of 100 Hz vibration was based on the sensing frequencies of mechanoreceptors, the Meissner Corpuscle and Pacinian Corpuscle. The Meissner Corpuscle can detect vibration ranged between 30 and 60 Hz. The Pacinian Corpuscle can detect vibration ranged between 60 and 300 Hz [11,12]. Also, research studies showed that a frequency higher than 125 Hz may cause vibration injury [12,23]. Thus, we chose one frequency (i.e., 35 Hz) for activating the Meissner Corpuscle and one frequency (i.e., 100 Hz) for activating the Pacinian Corpuscle. A laser Doppler flowmetry (PeriFlux 5000, Perimed, Las Vegas, NV, USA) was used to measure SBF at the first metatarsal head with a sampling rate of 32 Hz. Each experimental protocol consisted of a 10-min baseline measurement of SBF, 10-min vibration, and 10-min measurement of SBF immediately after vibration. Two successive protocols were separated by a washout period of 30 min to allow SBF recover to the baseline level. Example of skin blood flow signals in response to three frequencies of local vibrations (i.e., 0, 35, and 100 Hz) are shown in Figure 1.

### 2.3. Sample Entropy and Multiscale Entropy

The regularity degree of a time series has been commonly quantified by sample entropy [24] (*E_s_*), which is an improved version of approximate entropy (*A_s_*) [25]. *E_s_* is defined as the negative natural logarithm of the conditional probability that two sequences within a tolerance *r* for *m* points remain within the tolerance at the next point [24]. A smaller value of *E_s_* indicates a higher degree of regularity. Although *A_s_* and *E_s_* are frequently referred to as complexity measures of time series, they are actually measures of regularity. For instance, the *E_s_* algorithm assigns the largest value to white noise, which is totally unpredictable but possess no structural complexity. Also, it has been found that *E_s_* may yield lower values for physiological time series under healthy conditions compared to pathologic time series [26]. This observation does not agree with the concept that impaired systems generally lose their ability for adjustment, and thus their outputs exhibit less complexity [26]. In this context, several multiscale entropy (MSE) methods were introduced [27,28,29]. In the first MSE method proposed by Cost et al. [27], a set of coarse-grained time series are constructed by dividing the original time series into non-overlapping windows of length *τ* and taking the average of the data points inside each window as an element of the coarse-grained time series. Then, *E_s_* is calculated for each coarse-grained time series, which can be viewed as a function of *τ*. This method has several limitations. First, the procedure for constructing coarse-grained time series is similar to applying a low-pass filter to the original time series followed by a downsampling operation. Since the cutoff frequency is determined by *τ*, there may be situations in which the frequency components of the original time series attributed to a certain underlying mechanism are partially filtered out. On the other hand, this procedure has been found to produce artifacts [29]. Second, this method adopts a constant tolerance for all scales, while the standard deviation of the coarse-grained time series likely decreases with increasing scales, contributing to a decrease in the resultant entropy values at larger scales. Additionally, the length of the coarse-grained time series rapidly decreases with increasing scales, leading to unreliable entropy estimations at large scales, especially for short time series. For details of the limitations of the MSE method and several improved methods, see [16,17,30].

### 2.4. Modified Sample Entropy

In our previous study [21], a modified sample entropy algorithm was developed. Its procedures are presented briefly as follows. For a time series, {*x*(*i*), *i =* 1, …, *N*}, its *m*-point sequences are defined as:

(1) $$x_{m}^{\tau }(i)=\{x(i+k\tau ),0\leq k\leq m-1\},\;1\leq i\leq N-m\tau$$

where *τ* is a delay. The distance between two sequences $x_{m}^{\tau }(i)$ and $x_{m}^{\tau }(j)$ is defined as:

$$d[x_{m}^{\tau }(i),x_{m}^{\tau }(j)]=\max\{|x(i+k\tau )-x(j+k\tau )|,0\leq k\leq m-1\},$$

(2) 1≤i,j≤N−mτ, |j−i|>τ.

For a given sequence $x_{m}^{\tau }(i)$, suppose the number of $x_{m}^{\tau }(j)$, where |j−i|>τ, is $n_{i}$ and the number of $x_{m}^{\tau }(j)$ that matches $x_{m}^{\tau }(i)$, i.e., $d[x_{m}^{\tau }(i),x_{m}^{\tau }(j)]<r$ and |j−i|>τ, is $n_{i}^{m}(r)$, where r is a selected tolerance, usually being proportional to the standard deviation of the time series. Therefore, $C_{i}^{m}(r)=n_{i}^{m}(r)/n_{i}$ is the probability that any sequence $x_{m}^{\tau }(j)$ matches $x_{m}^{\tau }(i)$; and $C^{m}(r)=\sum_{i=1}^{N-m\tau }C_{i}^{m}/(N-m\tau )$ is the probability that any two sequences $x_{m}^{\tau }(i)$ and $x_{m}^{\tau }(j)$ are matched. Likewise; and $C^{m+1}(r)$ is the probability that any two sequences $x_{m+1}^{\tau }(i)$ and $x_{m+1}^{\tau }(j)$ are matched. The modified sample entropy is defined as:

(3) $$E_{ms}(m,r,\tau )=\lim_{N\to \infty }-\ln\frac{C^{m+1}(r)}{C^{m}(r)}$$

which is estimated by the statistic:

(4) $$E_{ms}(m,r,\tau ,N)=-\ln\frac{C^{m+1}(r)}{C^{m}(r)}$$

**Figure 2:**
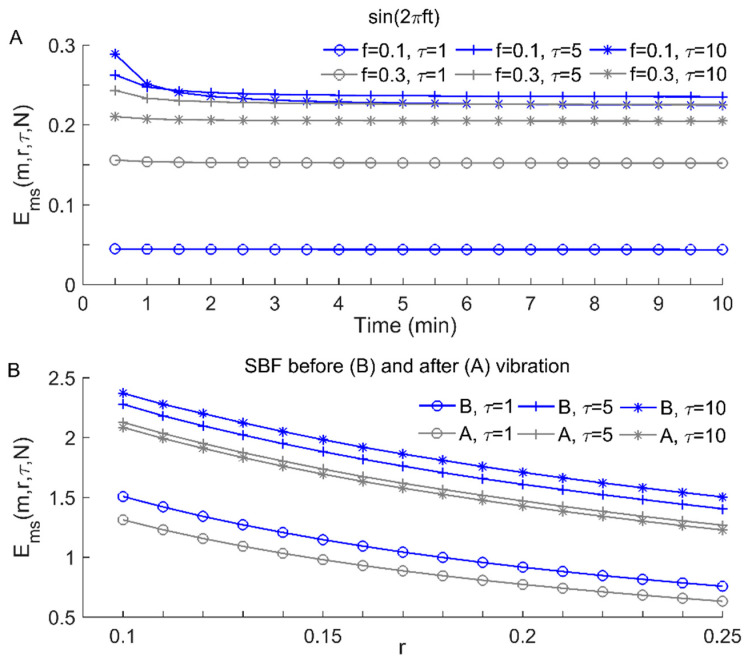
(**A**) Modified sample entropy $E_{ms}(m,r,\tau ,N)$ of 0.1 and 0.3 Hz sinusoidal signals with different time durations from 0.5 to 10 min (corresponding to 960 to 19,200 points sampled at 32 Hz), where *m* = 2 and *r* = 0.2. (**B**) $E_{ms}(m,r,\tau ,N)$ of the SBF signals shown in Figure 1E for different *r* values from 0.1 to 0.25, where *m* = 2 and *N* = 19,200.

The performance of *E_ms_* has been evaluated in our previous studies using both simulated time series and SBF data [16,17,21]. The results showed that *E_ms_* is insensitive to *m* and relative consistent for varying values of *r* [21]. Here, we further demonstrate that *E_ms_* is largely independent of the length of time series. As shown in Figure 2A, for sinusoidal signals of length *N*, when *N* exceeds a certain threshold, *E_ms_* yields almost identical values with the other parameters *m*, *r*, and *τ* being fixed. On the other hand, as shown in Figure 2B, although *E_ms_* decreases monotonically with increasing tolerance *r*, it always yields a lower value for the SBF signal after vibration compared to the SBF signal before vibration.

**Figure 3:**
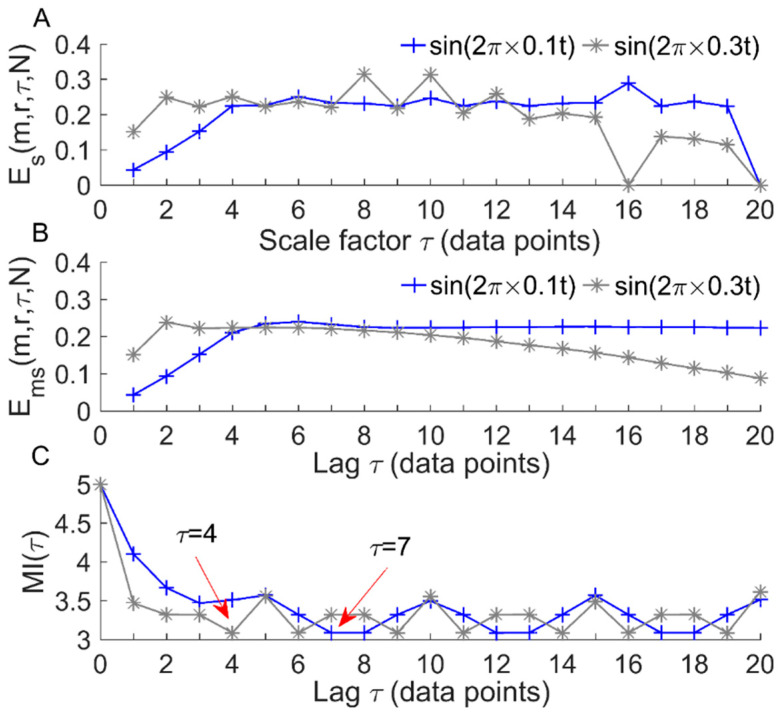
Multiscale entropy $E_{s}(m,r,\tau ,N)$ (**A**) and modified sample entropy $E_{ms}(m,r,\tau ,N)$ (**B**) for 0.1 and 0.3 Hz sinusoidal signals, where *m* = 2 and *r* = 0.2. The sampling rate (32 Hz) and length (19,200 data points) are identical to those of 10 min SBF signals, respectively. (**C**) Mutual information functions, MI(τ), of the sinusoidal signals. The first minimum of MI(τ) indicates that the optimal lags for sin(2π×0.1t) and sin(2π×0.3t) are *τ* = 7 and *τ* = 4, respectively if the regularity of the signals is quantified at a single scale.

When introducing multiple lags between successive data points of the original time series, i.e., multiple values of *τ*, *E_ms_* is actually a multiscale entropy measure [16,17]. As a supplement, here we further test the robustness of *E_ms_* and the MSE algorithm proposed by Costa et al. [27], denoted as *E_ms_*(*m*,*r*,*τ*,*N*), using 0.1 and 0.3 Hz sinusoidal signals. The motivations arise from the fact that the myogenic and neurogenic frequencies of SBF center approximately at 0.1 and 0.3 Hz, respectively, and that sinusoidal signals should yield the same entropy value no matter their frequencies as well as the parameters involved in the entropy measure. As shown in Figure 3, *E_ms_* gives almost identical values for 0.1 and 0.3 Hz sinusoidal signals for *τ* values from 4 to 10 (Figure 3B), whereas *E_s_* values of 0.3 Hz sinusoidal signal are unstable and can even be zero at larger scales due to the rapidly decreased signal length (Figure 3A). Note that when quantifying the regularity degree of a time series at a single scale using *E_ms_*, the optimal lag can be estimated by the first minimum of the mutual information function of the time series [21] (Figure 3C). If *τ* is significant lagging the optimal value, *E_ms_* may distinctly deviate from the expected value (Figure 3B). This observation suggests that when *E_ms_* is serving as a multiscale entropy, the lag should not be a significant lag from the optimal value. 

### 2.5. Application of E_ms_ to SBF Data

**Figure 4:**
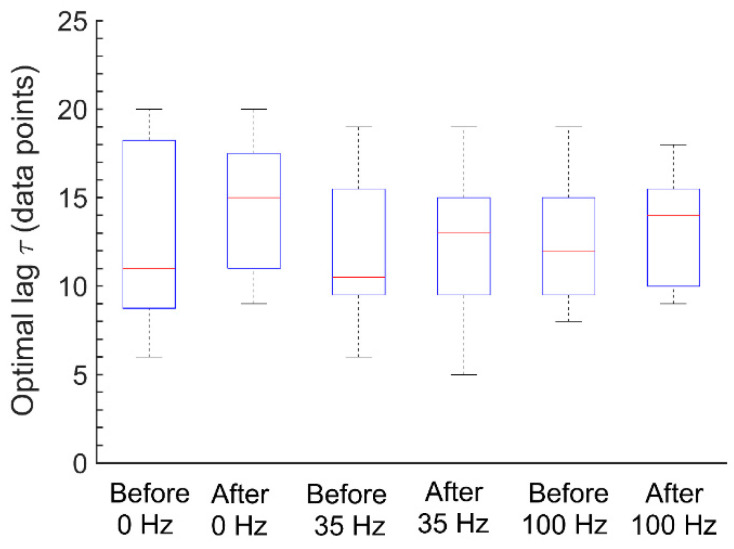
Boxplots of the optimal lags. For each SBF signal, the optimal lag was determined by the first minimum of the mutual information function of the signal.

We applied the *E_ms_* algorithm to SBF signals collected from 12 subjects before and after vibration at 0 Hz, 35 Hz, and 100 Hz. As noted earlier, SBF contains six characteristic frequencies between 0.005 and 2 Hz, including 0.005–0.0095, 0.0095–0.02, 0.02–0.05, 0.05–0.15, 0.15–0.4, and 0.4–2 Hz. Because the time duration of the SBF signal (10 min) may not be long enough to explore the lowest frequency (0.005–0.0095 Hz), we only considered five characteristic frequencies between 0.0095 and 2 Hz. Hence, each original SBF signal was filtered by decomposing it into a set of intrinsic mode functions using the ensemble empirical mode decomposition method [31] and accumulating the mode functions with frequency intervals between 0.0095 and 2 Hz. In order to choose a reasonable range of *τ* for all SBF signals, the optimal *τ* value for each signal was obtained from the first minimum of the mutual information function of the signal [21]. As shown in Figure 4, since these optimal values did not exceed 20, the range of *τ* was chosen as 1–20 data points. Therefore, we computed *E_ms_* for each filtered signal using the parameters *m* = 2, *r* = 0.2 × SD and *τ* = 1–20.

In order to further investigate how different frequencies of local vibration affect the regulatory mechanisms of SBF, we performed the following analyses. First, the SBF signals were filtered to remove the cardiac (0.4–2 Hz) and respiratory (0.15–0.4 Hz) oscillations while preserving metabolic (0.0095–0.02 Hz), neurogenic (0.02–0.05 Hz), and myogenic (0.05–0.15 Hz) oscillations through a similar procedure as described above. Then, we examined the mutual information function of each filtered signal aiming to choose a reasonable range of *τ*.

For most of the filtered signals, the mutual information function monotonously decreases very slowly in a wide range. Consequently, the value of *τ* corresponding to the first minimum can be very large, e.g., larger than 150 data points. We did not compute *E_ms_* for very large values of *τ*, because we observed that 100 Hz vibration induced a significant decrease in *E_ms_* at any scale, but 0 Hz and 35 Hz vibrations did not. Thus, we chose *τ* = 1–80 as the range of lags. Finally, we computed *E_ms_* for each filtered signal using the parameters *m* = 2, *r* = 0.2 × SD and *τ* = 1–80. 

For each vibration frequency, changes in *E_ms_* of SBF signals in response to vibration were examined using Wilcoxon signed-rank tests performed in SPSS 26 (SPSS, Chicago, IL, USA). The significance level was set at 0.05. 

## 3. Results

**Figure 5:**
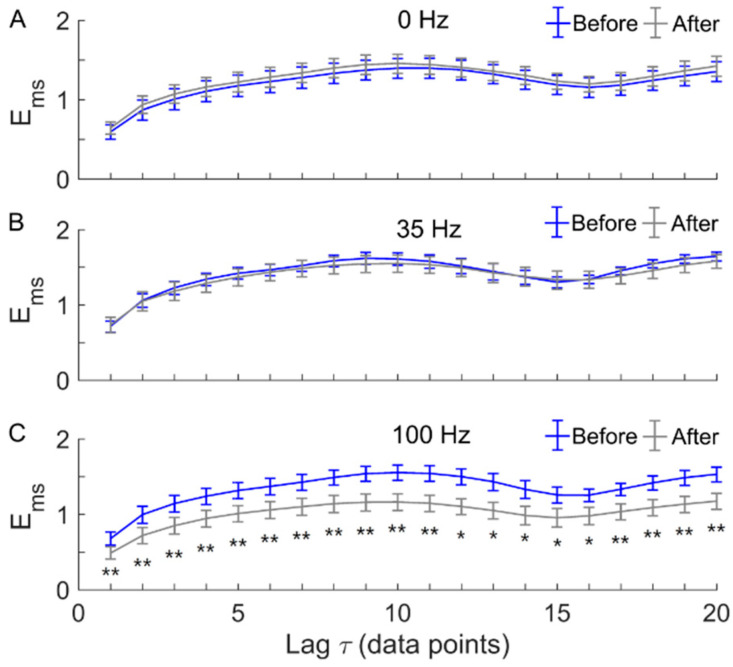
Multiscale entropy (*E_ms_*) of SBF signals before and after local vibration at 0 Hz (**A**), 35 Hz (**B**), and 100 Hz (**C**). Data are represented as mean ± standard errors. *E_ms_* showed little changes after 0 Hz or 35 Hz vibration (**A**,**B**) but a significant decrease after 100 Hz vibration (**C**). For 100 Hz vibration, *p* < 0.01 for *τ* from 1 to 11 and from 17 to 20, while *p* < 0.05 for *τ* from 12 to 16. ** *p* < 0.01; * *p* < 0.05.

Figure 5 shows multiscale entropy, *E_ms_*, of SBF signals before and after local vibration at three frequencies. The 100 Hz vibration induced significant decreases in *E_ms_* at all scales (Figure 5C), indicating more regular SBF after vibration. In contrast, 0 Hz or 35 Hz vibration did not lead to significant changes in *E_ms_* of SBF. 

**Figure 6:**
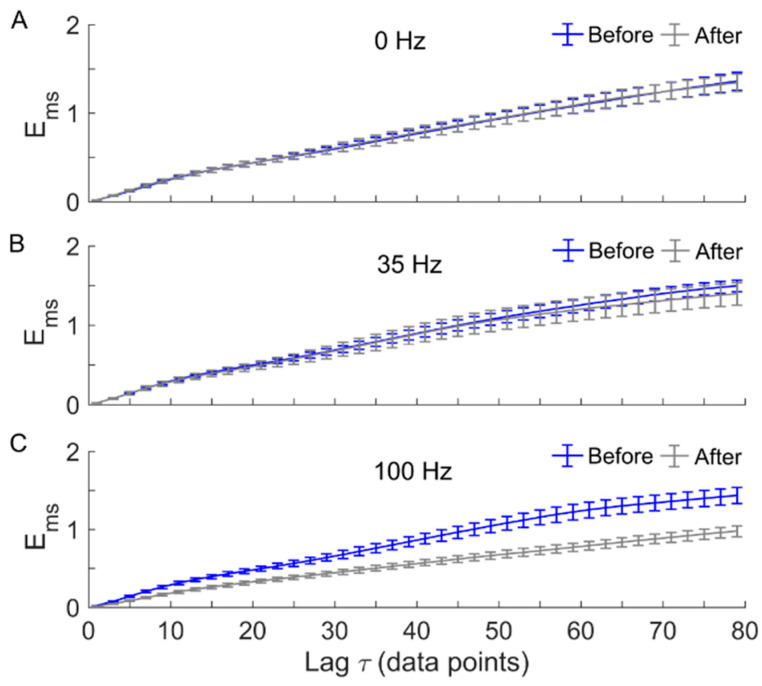
Multiscale entropy (*E_ms_*) of the filtered SBF signals (0.0095–0.15 Hz) before and after local vibration at 0 Hz (**A**), 35 Hz (**B**), and 100 Hz (**C**). Data are represented as mean ± standard errors. *E_ms_* shows little changes after 0 Hz or 35 Hz vibration (**A**,**B**) but a significant decrease after 100 Hz vibration (**C**). *p* < 0.05 for τ from 1 to 22 and *p* < 0.01 for τ from 23 to 79.

Figure 6 shows *E_ms_* of SBF containing only metabolic, neurogenic, and myogenic components before and after local vibration at three frequencies. Compared with the above case, *E_ms_* underwent more significant decreases at larger scales after 100 Hz vibration (Figure 6A), whereas *E_ms_* still did not show significant changes after 0 Hz or 35 Hz vibration (Figure 6A,B).

## 4. Discussion

The main findings of the present study are that a local vibration at 100 Hz significantly increased structural regularity of SBF at the first metatarsal head of healthy adults, but 0 Hz (sham vibration) and 35 Hz vibration did not. This is a significant finding because both 100 and 35 Hz vibrations can significantly increase SBF. Our result supports our hypothesis that local vibrations at different frequencies can cause different responses in SBF dynamics (e.g., multiscale regularity). Also, the significant enhancement of regularity of SBF was mainly attributed to enhanced regularity of the three frequency components of SBF, including 0.0095–0.02 Hz (metabolic control), 0.02–0.05 Hz (neurogenic control), and 0.05–0.15 Hz (myogenic control). These findings support the concept that the effect of local vibration on SBF depends on vibration frequency and that nonlinear properties of SBF should be considered when assessing the effectiveness of vibration on SBF. 

**Figure 7:**
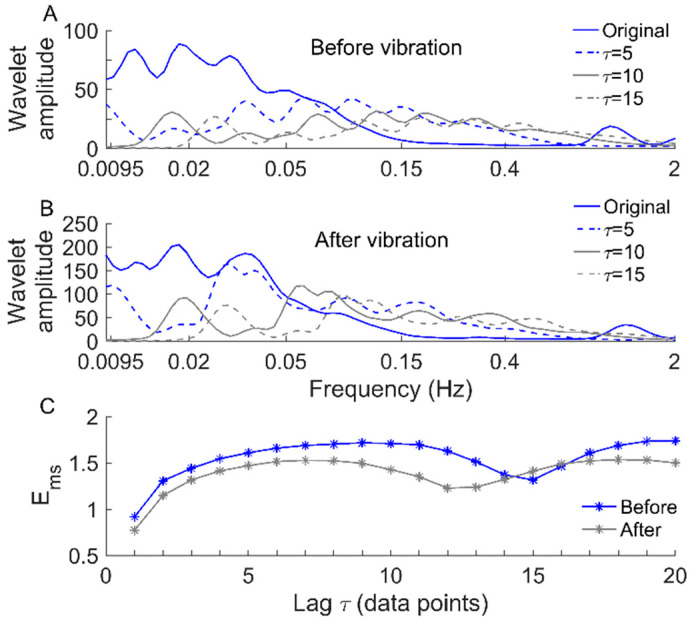
Wavelet amplitude spectra of the SBF signals shown in Figure 1E and newly constructed signals. (**A**) Wavelet amplitude spectra of the SBF signal before vibration and the constructed signals. (**B**) Wavelet amplitude spectra of the SBF signal after vibration and the constructed signals. (**C**) *E_ms_* for the SBF signals before and after vibration.

A modified sample entropy algorithm [21] was used to quantify the regularity of SBF in response to local vibration at multiple scales for the first time in this study. This was achieved by, for a SBF signal, introducing varying lags between successive data points of the sequences that are compared in the traditional sample entropy algorithm [24]. To understand how the lag *τ* affects *E_ms_*(*m,r,τ,N*), we performed the following experiment. Since computing *E_ms_*(*m*,*r*,*τ*,*N*) of a time series, {x(i),i=1,…,N}, is equivalent to constructing a new time series, $y^{\left(\tau \right)}$, and then computing the traditional sample entropy [24] of $y^{\left(\tau \right)}$, we examined the effect of τ on wavelet-based spectrum of $y^{\left(\tau \right)}$. In detail, $y^{\left(\tau \right)}=\{b_{1},\ldots ,b_{\tau }\}$, where $b_{i}=\{x(i),x(i+\tau ),\ldots ,x(i+k\tau )\}$, i=1,…,τ, and k is the maximal integer satisfying i+kτ≤N. When τ = 1, $y^{\left(\tau \right)}$ retrieves the original time series. Considering two SBF signals shown in Figure 1E, for each signal, we constructed $y^{\left(5\right)}$, $y^{\left(10\right)}$, and $y^{\left(15\right)}$ according to the above approach. As shown in Figure 7A,B, the wavelet amplitude spectrum of each SBF signal exhibits a few prominent peaks, while the spectra of $y^{\left(5\right)}$ and $y^{\left(10\right)}$ exhibit more but lower peaks. Correspondingly, *E_ms_*(*m*,*r*,*τ*,*N*) of each SBF signal monotonously increases with increasing *τ* from 1 to 5 (Figure 7C). However, for each SBF signal, although the spectrum of $y^{\left(10\right)}$ is more homogeneous than that of $y^{\left(5\right)}$ (Figure 7A,B), *E_ms_* yields almost equal values for them (Figure 7C). Moreover, for the SBF signal before vibration, despite the spectrum of $y^{\left(15\right)}$ is more homogeneous than that of $y^{\left(5\right)}$ (Figure 7A), *E_ms_* of $y^{\left(15\right)}$ is distinctly lower than that of $y^{\left(5\right)}$ (Figure 7C). These observations indicate that when *τ* varies from 1 to a small value, a larger value of *τ* leads to a more homogeneous combination of the frequency components of SBF, which contributes to a lager value of *E_ms_* (Figure 7C). When *τ* exceeds a certain range, larger values of *τ* do not necessarily lead to larger values of *E_ms_*. The possible reasons may be that larger lags do not necessarily lead to more homogeneous combinations of the frequency components of SBF and the homogeneity degree of SBF cannot be fully depicted by the wavelet-based spectrum. 

The choice of an appropriate scale range is an important issue for any multiscale entropy methods, which depends not only on the algorithm itself but also on the processed data. Our previous work suggested that when *E_ms_* serves as a single-scale entropy measure, the optimal lag could be determined by the first minimum of the mutual information function of the time series [21]. When *E_ms_* serves as a multiscale entropy measure applying to SBF signals, it usually rises with increasing scales in the initial stage and then reaches a plateau followed by a decrease. For most data sets used in the present study, *E_ms_* also shows such a trend (Figure 5). Typically, the optimal lag is around the end of the plateau stage of the *E_ms_* curve. For example, for the SBF singles shown in Figure 1E, the optimal lags are 10 (before vibration) and 11 (after vibration), respectively. From Figure 7C, it can be clearly seen that *τ* = 10 is close to the end of the plateau stage (before vibration), while *τ* = 11 (after vibration) is at the decreasing stage. Accordingly, we suggest that it would be reasonable to take the optimal lag as the upper limit of the scale range for computing *E_ms_*. Therefore, the scale range *τ* = 1–20 is large enough for computing *E_ms_* of the SBF signals. 

Our results showed that local vibration at 100 Hz caused a significant increase in the regularity of SBF (Figure 5C), and 0 Hz or 35 Hz vibrations did not affect the regularity of SBF (Figure 5A,B). Because there are no previous studies investigating the nonlinear properties of SBF in response to local vibrations, we were unable to directly compare our results with other studies [10,11,12]. Zhu et al. showed that SBF ratio, defined as the ratio of SBF after vibration to that before vibration, at the first metatarsal head of healthy adults was significant higher after 100 Hz vibration compared to that after 0 Hz or 35 Hz vibration [12]. Although the present study adopted the same experimental protocols, we focused on the changes in regularity of SBF in response to vibration at different frequencies rather than changes in magnitude of SBF. Nevertheless, our results support the use of a higher vibration frequency for improving SBF. However, there was a discrepancy between our observations and those reported by Zhu et al. [12] who observed a slight decrease and a mild increase in SBF ratio induced by 0 Hz and 35 Hz vibrations, respectively. This means that 0 Hz and 35 Hz vibrations induced changes in SBF ratio but not in regularity of SBF. 

The specific mechanisms responsible for our observations are unclear. One possible explanation is that different frequencies of vibration may activate different channels associated with mechanoreceptors in the plantar skin. It is known that the glabrous skin contains a large number of Pacinian corpuscles, which are more sensitive to higher vibration frequencies [23]. Thus, a higher frequency of vibration may be easier to induce SBF response [8]. Additionally, a higher vibration frequency may activate more mechanoreceptors, including Pacinian corpuscles and Messinian corpuscles [32], thereby contributing a more intense SBF response.

Our results also showed that when the SBF signal was filtered to preserve only metabolic (0.0095–0.02 Hz), neurogenic (0.02–0.05 Hz), and myogenic (0.05–0.15 Hz) components, *E_ms_* underwent a more prominent decrease after 100 Hz vibration compared to the original signals (Figure 6C), while 35 Hz and 0 Hz vibrations had no or little effect on *E_ms_* of the filtered SBF signals (Figure 6A,B). This observation implies that the enhancement of regularity of SBF induced by 100 Hz vibration was mainly attributed to enhanced regularity of the oscillatory components associated with the local control mechanisms. Our results were roughly consistent with the literature viewpoint that two main mechanisms, including the nitric oxide production and nerve axon reflex, are responsible for the increase in SBF in response to vibration [6]. Maloney-Hinds et al. reported that 50 Hz vibration delivered from a vibrating platform to the forearm of healthy adults and adults with type 2 diabetes induced increases in SBF and nitric oxide production [9]. Strzalkowski et al. showed that 150 Hz vibration applied to the hand palm and foot sole of healthy subjects produced reductions in burst occurrence of muscle sympathetic nerve activity [32]. Zhu et al. demonstrated that under 100 Hz vibration, ratios of wavelet amplitude of metabolic and neurogenic components of SBF, defined as the ratio of mean absolute wavelet coefficient over the frequency band and over time after vibration to that before vibration, were significantly higher compared to 0 Hz vibration [12]. The authors thus suggested that the increase in SBF induced by 100 Hz vibration was associated with metabolic and neurogenic controls. On the other hand, our results revealed distinctive features of SBF in response to vibration that cannot be depicted by linear approaches such as wavelet analysis. For instance, our results showed that under 100 Hz vibration, the decrease in *E_ms_* of the filtered SBF signals was more prominent compared to that of the original SBF signals (Figure 5C and Figure 6C). On the contrary, in the study by Zhu et al. [12], the difference in the ratio of wavelet amplitude for either the metabolic or neurogenic oscillation between 100 Hz and 0 Hz vibrations was less significant than the difference in the SBF ratio between two vibration frequencies. Therefore, structural properties of SBF in response to vibration should be considered when assessing the efficacy of vibration on SBF.

This study has several limitations. First, we recruited only 12 participants. The small sample size might impede a reliable statistical analysis. However, the main purpose of this study was to investigate whether regularity of SBF can be used to characterize SBF response to vibration. Our results showed that this measure underwent distinctively different changes after vibration at different frequencies, suggesting it could play an important role in selecting vibration parameters. Second, only three vibration frequencies, i.e., 100 Hz, 35 Hz, and 0 Hz (sham vibration) were tested in this study. It is unclear whether vibration at a mild frequency, e.g., 50 Hz, could induce a significant change in regularity of SBF. Third, previous studies suggested that other parameters of vibration, such as the intensity of vibration (e.g., amplitude of vibration) also influence SBF response [6], which were not considered in this study. Their influences of vibration intensity on SBF response need to be examined in future studies. 

## 5. Conclusions

The main findings of the present study are that local vibration at 100 Hz significantly increased structural regularity of SBF at the first metatarsal head of healthy adults, but 0 Hz (sham vibration) and 35 Hz vibration did not. This is a significant finding because both 100 and 35 Hz vibrations can significantly increase SBF. Our result supports our hypothesis that local vibrations at different frequencies can cause different responses in SBF dynamics (e.g., multiscale regularity). These findings support the concept that the effect of local vibration on SBF should be assessed using both traditional time- and frequency-domain and multiscale regularity methods.
